# A Train-the-Trainer Approach to Build Community Resilience to the Health Impacts of Climate Change in the Dominican Republic

**DOI:** 10.3390/ijerph22040650

**Published:** 2025-04-20

**Authors:** Hannah N. W. Weinstein, Kristie Hadley, Jessica Patel, Sarah Silliman, R. Yamir Gomez Carrasco, Andres J. Arredondo Santana, Heidi Sosa, Stephanie M. Rosa, Carol Martinez, Nicola P. Hamacher, Haley Campbell, James K. Sullivan, Danielly de Paiva Magalhães, Cecilia Sorensen, Ana Celia Valenzuela González

**Affiliations:** 1 Global Consortium on Climate and Health Education, Columbia University, New York, NY 10032, USAcjs2282@cumc.columbia.edu (C.S.); 2 Vagelos College of Physicians and Surgeons, Columbia University, New York, NY 10032, USA; 3 Department of Emergency Medicine, Columbia Irving Medical Center, New York, NY 10032, USA; 4 Columbia World Projects, Columbia University, New York, NY 10027, USA; 5 Department of Environmental Health Sciences, Mailman School of Public Health, Columbia University, New York, NY 10032, USA; 6 Instituto de Medicina Tropical y Salud Global, Universidad Iberoamericana Santo Domingo, Santo Domingo 22333, Dominican Republic; a.valenzuela1@unibe.edu.do

**Keywords:** climate change, health education, climate resilience, community resilience, capacity building, train-the-trainer course series

## Abstract

Communities in the Dominican Republic (DR) face increased natural disasters, poor air quality, food insecurity, and health impacts related to climate change. We evaluated the success of a train-the-trainer program to empower community leaders, women, and at-risk youth with the knowledge and skills to increase individual and community resilience in Cristo Rey, Dominican Republic. Three in-person two-day courses were conducted between July and August 2024 at the Universidad Iberoamericana. Each session included eight lectures and collaborative learning activities on climate change science, adaptation, resilience, and health impacts. Intra-group analyses comparing pre- and post-course surveys assessed participants’ climate change awareness, literacy, and communication and response skills. One hundred and four attendees participated in the survey study. Of the 100 participants with demographic data, 55% (*n* = 55) were 35 years old or younger, 70% (*n* = 70) identified as female, and 45% (*n* = 45) lived in Cristo Rey. The participants reported high baseline climate change awareness. Compared to before the course, the participants reported increased literacy regarding the environmental impacts of climate change relevant to the DR and the specific health impacts (*p*-value < 0.05) and increased climate change-related communication and response skills (*p*-value < 0.001). This study suggests competency-based, regional-specific courses deployed in a train-the-trainer model, have the potential to equip community members with knowledge to protect their health.

## 1. Introduction

The adverse effects of climate change are now apparent and present urgent and complex challenges to human health and health systems globally. Heat waves, rainstorms, and flooding are becoming more deadly; disease outbreaks (dengue, malaria, cholera, etc.) last longer and are seen in new regions; wildfires release stored carbon and reduce air quality; and food and water security are threatened by drought and extreme weather. On an individual and community level, changing climatic patterns threaten the tenets of a stable and secure livelihood, resulting in deteriorating mental health, forced migration, and civil instability. Island nations, such as the Dominican Republic (DR), are disproportionately vulnerable to the impacts of climate change while contributing minimally to the generation of fossil fuel pollution [1,2,3,4]. The increase in extreme weather events and slow-onset climatic changes, such as sea level rise, has forced many communities across the DR to adapt and strengthen their physical infrastructure, adjust policies and plans, and create new mechanisms for communities to better prepare and respond [5,6,7,8]. Climate change and extreme weather in the DR are significantly impacting community health, with impacts projected to worsen in future low- or high-emission scenarios [9]. Despite the existence of a National Climate Change Policy, the integration of climate resilience into public health planning and budgeting remains limited [10]. Strengthening public health systems’ capacity to respond to climate-related health threats through the three-tiered private, public, and subsidized healthcare model requires intersectoral coordination, investment in climate-informed health planning, community-based preparedness, and dedicated funding streams for adaptation and emergency response.

As with many communities across the DR, Cristo Rey, a neighborhood of Santo Domingo, is at risk of multiple acute and chronic infectious and non-communicable diseases that have become more severe because of increased hurricanes, flooding, food insecurity, exposure to extreme heat, and air pollution [9,11]. Despite facing challenges ranging from crowded living conditions and high density of informal housing to unemployment, the community of Cristo Rey has a long history of entrepreneurship and community engagement and maintains a rich culture and identity [12]. Given the high social and structural vulnerability of the community, facilitating community resilience to the health impacts of climate change is of particular importance.

Here, we describe the development and implementation of the initiative, Curso de Primeros Auxilios Comunitarios sobre el Clima y las Catástrofes en la Resiliencia Comunitaria, a climate change and resilience train-the-trainer program for health professionals, and a parallel training course for community members of Cristo Rey and nearby areas of Santo Domingo. The primary aims of this program were to (1) empower community leaders, women, and at-risk youth with the knowledge and skills to increase individual and community resilience and (2) develop and implement a program to increase the number of health professionals who are competent in and capable of providing high-quality and engaging climate and health education to community members. Here, we report the evaluation of the effectiveness of aim 1: a two-day workshop to improve community knowledge of climate and health risks and protective strategies. The evaluation assessed three types of skills: (1) climate change and health awareness, (2) climate change literacy, and (3) communication and response skills. To our knowledge, this is the first study focused on improving community resilience to the health risks of climate change through a train-the-trainer model.

## 2. Materials and Methods

This initiative was designed by the Global Consortium of Climate and Health Education (GCCHE) as part of the Columbia World Project (CWP) “Building Climate Resilient Communities in the Dominican Republic” program, in collaboration with the Instituto de Medicina Tropical & Salud Global—Universidad Iberoamericana (UNIBE), ProFamilia, the Red Cross, Hospital General de la Plaza de la Salud, and Alcaldía del Distrito Nacional [12]. The study and survey were approved by the UNIBE Institutional Review Board. The initiative consisted of two parallel programs: (1) a train-the-trainer program and (2) community training.

### 2.1. Train-the-Trainer Program

The program was based on a train-the-trainer model, with the aim of increasing the number of health professionals who are competent in and capable of providing high-quality and engaging climate and health education to community members in the DR. The trainers consisted of four clinical health professionals from Columbia University Irving Medical Center (CUIMC), and the trainees consisted of six health professionals with two each from UNIBE, ProFamilia, and Hospital General de la Plaza de la Salud. The trainees represent a mix of academic, public, and non-governmental health institutions in the Dominican Republic: UNIBE is a private academic institution involved in health research and education; Profamilia is a well-established non-governmental organization focused on community health services; and the Hospital General de la Plaza de la Salud is a leading public–private healthcare center that provides specialized medical services and public health programs. The CUIMC climate and health experts developed the initial course curriculum and content (slide decks, speaker notes, small group cases/activities) and, through collaborative sessions, trained the DR professionals. The collaborative training sessions were structured to gradually build the local health professionals’ knowledge and confidence. Over two months, April–May 2024, the trainers and trainees met weekly to review and discuss the course curriculum and materials, ensuring that it was well adapted to the local context. Based on feedback from the DR health professionals, the course was updated to include localized terminology and resources.

### 2.2. Community Training

The community course consisted of two-day in-person sessions, totaling 14–16 h. The course was taught in Spanish and offered three times between 5 July and 24 August 2024, at UNIBE in Santo Domingo, DR. There was no charge for community members to participate and transportation from the community of Cristo Rey to UNIBE was provided. The course curriculum was based on GCCHE core competencies in climate and health for health professionals and was created in collaboration with the trainers and trainees [13,14,15]. The course included eight lectures and eight associated collaborative learning activities with content and resources specific to the DR (Appendix A, Table A1). The interactive lectures addressed key topics, including climate change science, climate adaptation and resilience, and the health impacts of heat, air pollution, vector-borne illness, extreme weather events, disasters, and community mental health. The eight collaborative learning activities included discussion questions, action plan development, conceptual maps, and mock scenarios. Slides and printed workbooks were provided to each participant. Following the course, all the participants were offered free cardiopulmonary resuscitation (CPR) and first aid training through the Red Cross.

The first workshop was delivered by the CUIMC experts, with the DR health professionals observing. The second workshop was offered by a mix of the CUIMC experts and DR health professionals, and the final session was conducted exclusively by the DR health professionals with support from the program team. This approach ensured a seamless transition to local leadership in delivering the training.

### 2.3. Recruitment and Enrollment

This educational initiative was designed for the community members of Santo Domingo, specifically the Cristo Rey neighborhood. The aim was to engage 90+ at-risk youth and women from the Cristo Rey area, as well as community leaders and interested residents from the city at large. Participants were invited through outreach via social media, phone calls, and door-to-door contact organized by members of ProFamilia and Junta de Vecinos, an organization in Cristo Rey. Each participant over the age of 18 was invited to enroll in the survey study. Participation in the survey and study was voluntary, and each participant provided informed consent.

### 2.4. Survey and Evaluation

The study included pre- and post-course surveys developed by the GCCHE, CWP, and UNIBE (Appendix A, Table A2). The pre-course survey consisted of 15 multiple-choice questions, assessing the participants’ awareness of climate change, climate change literacy, and communication and response knowledge and skills. The post-course survey consisted of the same 15 questions, as well as 11 questions asking the participants to evaluate the quality and applicability of the course. For the first session, data were collected electronically on the participants’ phones or computers using the Qualtrics system. For the second and third sessions, data were collected on a printed questionnaire, with each question projected on a large screen and read out loud in Spanish. The survey data were then manually entered into the Qualtrics system by two investigators. Deidentified data were extracted separately following each session and merged for analysis. The Strengthening the Reporting of Observational Studies in Epidemiology (STROBE) checklist criteria were followed.

### 2.5. Statistical Analysis

All the data were compared using R version 4.4.0 in September 2024. Statistical analyses included intra-group analyses comparing data from the pre-course and post-course surveys. Each participant’s “baseline” was defined as the pre-course survey. Binary and categorical variables were compared using Fisher’s exact tests to assess change in the proportions of the survey participants’ responses from pre- to post-course. A *p*-value of less than 0.05 was considered significant.

## 3. Results

### 3.1. Demographics and Participation

**Figure 1:**
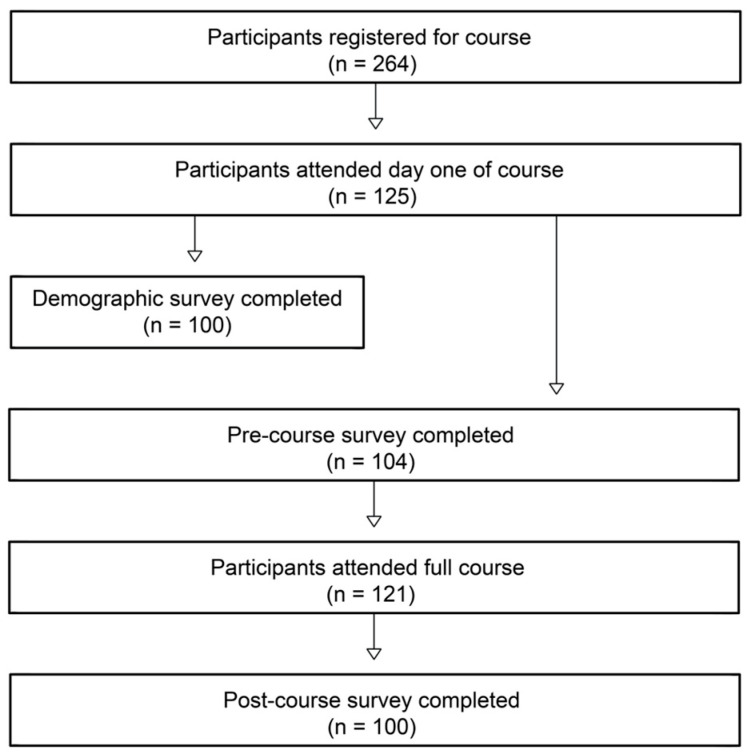
Course participation and survey completion. An initial 264 community members registered for the course, with 125 attending one day and 121 attending both days of one of the sessions. Of the individuals in attendance who were ≥18 years old, 104 completed the pre-course survey and 100 completed the post-course survey.

Over the three sessions, there were 264 enrollees with 121 in attendance for the full two-day course (Figure 1). Of the 100 course participants with complete demographic data, 10% (*n* = 10) were between 15 and 17 years old, 24% (*n* = 24) were between 18 and 24 years old, 21% (*n* = 21) were between 25 and 35 years old, and 45% (*n* = 45) were above 35 years old. In total, 70% (*n* = 70) of the subjects identified as female. Regarding the place of residence in Santo Domingo, 49% (*n* = 49) lived in Cristo Rey. Of the participants in attendance who were ≥ 18 years old, 104 completed the pre-course survey and 100 completed the post-course survey. Table 1 summarizes the general demographic data.

### 3.2. Climate Change Knowledge and Awareness

Baseline awareness of climate change was high in this cohort (Table 2). In the pre-course survey, 99% of the participants believed that climate change was real. When the participants were asked how concerned they were regarding climate change, 72% reported “very worried” before the course. Most (96%) of the participants stated that climate change affected their community before the course.

**Figure 2:**
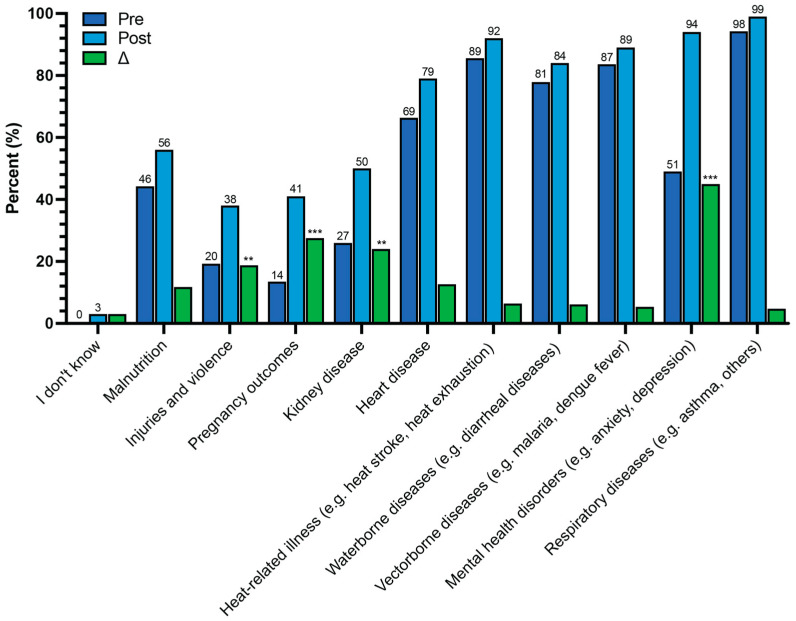
Percentage distribution of pre- and post-course responses and the percentage change regarding knowledge about climate change-related health issues. Responses to “If you answered yes to the previous question, which health problems do you think could be influenced by climate change? (Check all that apply).” *p*-value: “I don’t know” = 0.116; “Malnutrition” = 0.123; “Injuries and violence” = 0.003; “Pregnancy outcomes” < 0.001; “Kidney disease” = 0.001; “Heart disease” = 0.059; “Heat-related illness (e.g., heat stroke, heat exhaustion)” = 0.185; “Water-borne diseases (e.g., diarrheal diseases)” = 0.290; “Vector-borne diseases (e.g., malaria, dengue fever)” = 0.312; “Mental health disorders (e.g., anxiety, depression)” < 0.001; “Respiratory diseases (e.g., asthma, others)” = 0.119. Bars represent the percentage of respondents selecting each answer choice. Numbers represent the number of respondents selecting each answer choice. “Pre” = pre-course survey responses; “Post” = post-course survey responses. ** *p*-value < 0.01; *** *p*-value < 0.001.

After the course, the number of participants reporting that climate change meant changes in weather patterns, sea level, temperature, the environment, extreme heat, and global warming increased (Table 2). At the start, 60% of the participants answered that climate change meant “extreme heat” to them; this significantly increased by 17% to 77% of the participants after the course (*p*-value = 0.010). The increases were not statistically significant for the other topics (*p*-value > 0.05). Only 50% (*n* = 52) of the participants answered that climate change meant changes in sea level, with the response rate increasing to 58% (*n* = 58) after the course (*p*-value = 0.264). The text continues here (Figure 2 and Table 2).

### 3.3. Climate Change Literacy

Compared to before the course, participants identified significantly more of the impacts of climate change relevant to the DR (Table 2). After the course, 27% more identified rising sea levels (*p*-value < 0.001), 18% more identified stronger hurricanes (*p*-value = 0.008), 17% more identified droughts (*p*-value = 0.015), and 16% more identified poorer air quality (*p*-value = 0.019). Flooding and higher temperatures were identified by most participants before and after the course, while few selected melting glaciers as relevant to the DR. No participants selected colder winters as a climate change impact relevant to the DR.

Regarding the acuity and cause of climate change, most participants reported that climate change was happening now and was at least partially due to human activities (Table 2). For instance, the majority (94%) acknowledged that climate change was happening now, while few (6%) thought it would happen in the future. Many participants (51%) thought that climate change was mainly or totally due to human activities, while approximately a third (38%) responded that climate change was due to both human activities and natural environmental changes. These percentages did not significantly change after the course.

The participants had high pre-course knowledge of the effects of climate change on health (Table 2). For instance, more than 80% responded that climate change affected them personally, their community, their country, other countries, and future generations either “moderately” or “a lot”. Additionally, before the course, 100% of the participants acknowledged that climate change affected human health, and 90% of the participants believed it was “very important” for individuals to understand how climate change affects health. Compared to before the course, the participants significantly identified more of the health impacts related to climate change (Figure 2). For instance, of the health risks influenced by climate change, after the course 45% more identified mental health disorders (e.g., anxiety, depression) (*p*-value < 0.001), 28% more identified pregnancy outcomes (*p*-value < 0.001), 23% more identified kidney disease (*p*-value = 0.001), and 19% more identified injuries and violence (*p*-value = 0.003). The participants’ pre-course knowledge was high (>80%) for heat-related illnesses, waterborne diseases, vector-borne diseases, and respiratory diseases.

### 3.4. Communication and Response Skills

**Figure 3:**
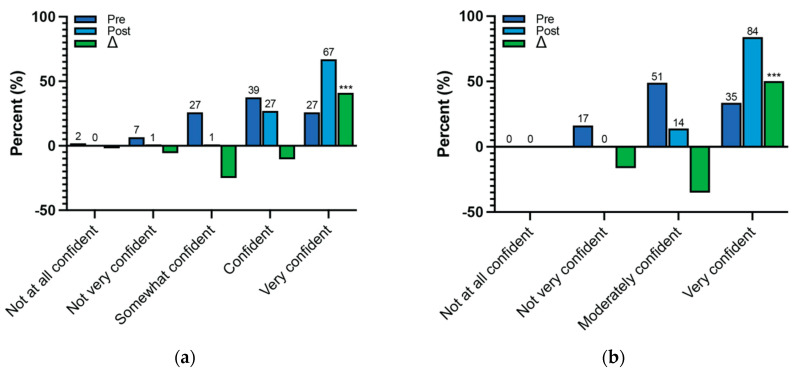

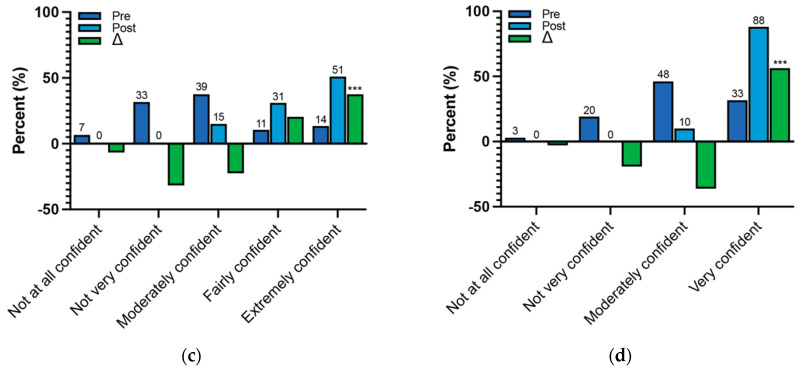
Percentage distribution of pre- and post-course responses and the percentage change regarding communication and response skills. (**a**) Responses to “How confident do you feel talking to others about how climate change affects their health?” Pre-course: *n* = 102; post-course: *n* = 96. *p*-value < 0.001. (**b**) Responses to “How confident do you feel in your ability to communicate actions that community members can take to prevent climate-related health effects?” Pre-course: *n* = 103; post-course: *n* = 98. *p*-value < 0.001. (**c**) Responses to “How confident are you that you can protect yourself, your family, and your community from climate-related health problems?” Pre-course: *n* = 104; post-course: *n* = 97. *p*-value < 0.001. (**d**) Responses to “How confident do you feel in your ability to identify individuals and populations at risk for climate-related health effects?” Pre-course: *n* = 104; post-course: *n* = 98. *p*-value < 0.001. Bars represent the percentage of respondents. Numbers represent the number of respondents. “Pre” = pre-course survey responses; “Post” = post-course survey responses. *** *p*-value < 0.001.

The number of participants who reported feeling confident in their ability to communicate with others regarding how climate change affects their health and the actions that community members can take to prevent climate-related health effects increased significantly after the course (Figure 3). At the end, 41% more participants felt “very confident” discussing and clearly explaining how climate change impacts health (*p*-value < 0.001) (Figure 3a). Additionally, at the end, 50% more participants felt “very confident” about communicating actions that community members can take to prevent climate-related health effects (*p*-value < 0.001) (Figure 3b).

Compared to before the course, the number of participants who reported feeling confident in their ability to protect their family and in their capacity to identify populations at risk of climate-related health outcomes significantly increased (Figure 3). At the end, 38% more participants felt “extremely confident” in protecting their family and community from climate-related health outcomes (*p*-value < 0.001) (Figure 3c). Finally, at the end, 56% more participants felt “very confident” in their ability to identify individuals and populations at risk of climate-related health outcomes (*p*-value < 0.001) (Figure 3d).

## 4. Discussion

Health professionals and communities stand on the front lines of the climate crisis, yet many barriers prevent engagement and meaningful action to mitigate and adapt. Rapid knowledge dissemination, capacity building, and actions of health professionals are needed to protect patients, communities, and health systems. This program was successful in its two aims: to empower community leaders, women, and at-risk youth with the knowledge and skills to increase individual and community resilience and to increase the number of health professionals who are competent in and capable of providing high-quality and engaging climate and health education to community members. To our knowledge, this is the first study to evaluate the effectiveness of a train-the-trainer model to build capacity simultaneously among health professionals and community members related to the climate crisis.

Pre-course climate change awareness was high in this cohort, with most participants reporting knowledge about the existence, acuity, concerns, causes, and impacts of climate change in the DR. Yet, post-course evaluation demonstrated significant improvement in the participants’ literacy about the health impacts of climate change, notably identification of the fact that climate change may increase injuries and violence, pregnancy outcomes, kidney disease, and mental health disorders. Additionally, after the course, the participants reported significant improvements in all four questions assessing communication and response skills relevant to the health impacts of climate change, a topic previously highlighted as a priority in capacity building initiatives [16,17,18]. Overall, these findings confirm the course’s improved community—which included mainly youth (55%) and females (70%)—knowledge about the health impacts of climate change and effective response.

While this program is the first of its kind in the DR, it builds off previous courses implemented in Latin America and the Caribbean. In an 8-week virtual training course focused on climate and health topics related to the Pan American Health Organization region, participating health professionals reported increased climate and health communication, climate knowledge incorporation in professional practice, and confidence in engaging in climate initiatives [15]. Following a mixed in-person and virtual course specific to the Bahamas and a virtual course specific to Brazil, healthcare professionals and community members reported increased climate change and health knowledge [14,19]. Additionally, in a virtual 10-week climate and health course focused on impacts in the Caribbean, health professionals reported increased communication, engagement, and application of climate and health knowledge and skills after the course [20]. Taken together, these courses highlight the utility of climate and health education in improving individual and community preparedness to address the health risks of a changing climate. However, longer-term follow-up studies are needed to understand how knowledge translates into actual improvements in health outcomes at individual and community levels.

Courses that improve the health profession and community resilience are essential given the increasing climate change-related health hazards in the DR. Compared to 1981–2010, the World Health Organization estimates that temperatures in the DR will increase by approximately 3.2 °C with the annual percentage of hot or “heat stress” days increasing from 10% to 95% by the 22nd century in a high-emissions scenario—which is the current global trajectory given that energy-related CO_2_ emissions reached an all-time high in 2023 [1,9]. The increasing heat exposure, along with the increasing precipitation, intensity of hurricanes, and sea level rise, expose DR citizens to a risk of heat stress, vector-borne diseases, and malnutrition, among other consequences [9]. While the DR healthcare system has the capacity to respond to climate-related events, no formal national climate and health curriculum exists for health professionals [9]. This train-the-trainer course prepared six local healthcare professionals to be leaders in climate and health education specific to the DR while training youth and women to build cross-generational and gender-equitable resilience. Further initiatives and sustainable funding are necessary to expand training to additional regions of the DR. Future courses can utilize the expertise of the DR-based trainers to facilitate new courses and to train more leaders among health professionals.

### Strengths and Limitations

This program has multiple strengths. First, there was the high enrollment and engagement of youth and women, which exceeded the original recruitment goal of 90, allowing further education of potentially vulnerable populations. Second, the project facilitated pathways for future climate and health projects by creating multi-organization collaborative partnerships within the DR. There was deep engagement of health professionals within the DR who served as course faculty, allowing the expansion of local knowledge and making it possible for subsequent iterations of the course to be led entirely by local healthcare professionals. There were also opportunities for bi-directional engagement among course faculty and participants, improving the relevance and specificity of the content. Finally, this project utilized an evidence-based approach which adhered to global competencies for health professionals in responding to the climate and health crisis to ensure the content was appropriate and up-to-date.

An important limitation to recognize is the self-selection of participants into the course and into the study cohort. The results are therefore vulnerable to selection bias as enrollment and participation were voluntary and non-random. Thus, the results may not reflect the knowledge of the general population in Santo Domingo. Given that participants who were already interested in climate change may have been more likely to participate, this could explain the high pre-course climate change awareness.

## 5. Conclusions

Climate change is a reality in the Dominican Republic that will continue to have a significant impact on human health and the health sector. Health professionals and communities are on the front lines of impacts and occupy a critical position in the response to climate change, including climate mitigation and adaptation. Train-the-trainer programs have the potential to equip new cadres of health professionals to become essential health messengers within communities while simultaneously engaging those within communities, including youth and women, who are often at the highest risk of negative impacts.

## Figures and Tables

**Table 1:** Baseline participant demographics.

	Session 1 (*n* = 21)	Session 2 (*n* = 38)	Session 3 (*n* = 41)	Total (*n* = 100)
Age (years)—No. (%)				
15–17	0 (0)	5 (13)	5 (12)	10 (10)
18–24	2 (10)	11 (29)	11 (27)	24 (24)
25–35	4 (19)	7 (18)	10 (24)	21 (21)
35+	15 (71)	15 (40)	15 (37)	45 (45)
Female (sex)—No. (%)	12 (57)	23 (61)	35 (85)	70 (70)
Neighborhood (Cristo Rey)—No. (%)				
Since before 1 year old	1 (5)	1 (3)	1 (2)	3 (3)
Since 1–5 years old	0 (0.0)	4 (10)	2 (5)	6 (6)
Since 6–10 years old	1 (5)	1 (3)	6 (15)	8 (8)
Since 11–20 years old	2 (9)	3 (8)	0 (0)	5 (5)
Since >20 years old	11 (52)	15 (39)	1 (2)	27 (27)
I don’t live in Cristo Rey	6 (29)	14 (37)	31 (76)	51 (51)

**Table 2:** Climate change awareness and literacy among course participants.

Climate Change Awareness				
Do you believe that climate change is real?—No. (%)				
Response	Pre-Course Survey (n = 104)	Post-Course Survey (n = 100)	Percent Δ (%)	*p*-value
Yes	103 (99)	100 (100)	1	0.445
No	0 (0)	0 (0)	0	
I don’t know	1 (1)	0 (0)	−1	
How concerned are you about climate change?—No. (%)				
Response	Pre-course survey (n = 104)	Post-course survey (n = 100)	Percent Δ (%)	*p*-value
Very concerned	75 (72)	88 (88)	13	0.264
Somewhat concerned	26 (25)	10 (10)	−16	
Not concerned	1 (1)	2 (2)	1	
I don’t know, not sure	2 (2)	0 (0)	−2	
Do you think climate change is affecting your community?—No. (%)				
Response	Pre-course survey (n = 104)	Post-course survey (n = 100)	Percent Δ (%)	*p*-value
Yes	100 (96)	96 (96)	0	0.451
No	3 (3)	2 (2)	−1	
I don’t know	1 (1)	2 (2)	1	
What does “climate change” mean to you? (Check all that apply)—No. (%)				
Response	Pre-course survey (n = 104)	Post-course survey (n = 100)	Percent Δ (%)	*p*-value
Changes in weather patterns/conditions (e.g., more rainfall, more droughts)	78 (75)	80 (80)	5	0.408
Changes in sea level	52 (50)	58 (58)	8	0.264
Changes in temperature	79 (76)	81 (81)	5	0.400
Changes in the environment	72 (69)	78 (78)	9	0.204
Extreme heat	62 (60)	77 (77)	17	0.010
Global warming	76 (73)	77 (77)	1	0.628
**Climate change literacy**				
What climate change impacts are relevant to the Dominican Republic? (Check all that apply)—No. (%)				
Response	Pre-course survey (n = 104)	Post-course survey (n = 100)	Percent Δ (%)	*p*-value
More flooding	95 (91)	93 (93)	2	0.796
Stronger hurricanes	66 (63)	81 (81)	18	0.008
More droughts	55 (53)	70 (70)	17	0.015
Higher temperatures	84 (81)	90 (90)	9	1.000
Colder winters	0 (0)	0 (0)	0	1.000
Rising sea levels	44 (42)	69 (69)	27	<0.001
More forest fires	60 (58)	55 (55)	−3	0.778
Poorer air quality	67 (64)	80 (80)	16	0.019
Extinction of animal/plant species/loss of biodiversity	51 (49)	54 (54)	5	0.487
Melting glaciers	16 (15)	24 (24)	9	0.158
If climate change is real, when do you think it will happen?—No. (%)				
Response	Pre-course survey (n = 101)	Post-course survey (n = 98)	Percent Δ (%)	*p*-value
Happening now	95 (94)	96 (98)	4	0.462
In the future	6 (6)	2 (2)	−4	
I don’t know	0 (0)	0 (0)	0	
Assuming climate change is occurring, what do you think is causing it?—No. (%)				
Response	Pre-course survey (n = 104)	Post-course survey (n = 100)	Percent Δ (%)	*p*-value
Totally due to human activities	28 (27)	33 (33)	6	0.977
Mainly by human activities	25 (24)	21 (21)	−3	
Due to both human activities and natural environmental changes	40 (38)	37 (37)	−1	
Mainly due to natural environmental changes	4 (4)	6 (6)	2	
Totally due to natural environmental changes	7 (7)	3 (3)	−4	
None of the above because climate change is not happening	0 (0)	0 (0)	0	
How much do you think climate change affects the following? —No. (%)				
Response	Pre-course survey (n = 102)	Post-course survey (n = 98)	Percent Δ (%)	*p*-value
You personally				
None	1 (1)	2 (2)	1	0.808
A little	8 (8)	6 (6)	−2	
Moderately	37 (36)	36 (37)	1	
A lot	56 (55)	53 (54)	−1	
I don’t know	0 (0)	1 (1)	1	
Response	Pre-course survey (n = 100)	Post-course survey (n = 95)	Percent Δ (%)	*p*-value
People in your community				
None	0 (0)	3 (3)	3	0.105
A little	4 (4)	0 (0)	−4	
Moderately	22 (22)	13 (14)	−8	
A lot	69 (69)	76 (80)	11	
I don’t know	5 (5)	3 (3)	−2	
Response	Pre-course survey (n = 101)	Post-course survey (n = 93)	Percent Δ (%)	*p*-value
People in your country				
None	0 (0)	1 (1)	1	0.345
A little	0 (0)	1 (1)	1	
Moderately	12 (12)	4 (4)	−8	
A lot	87 (86)	86 (93)	7	
I don’t know	2 (2)	1 (1)	−1	
Response	Pre-course survey (n = 98)	Post-course survey (n = 93)	Percent Δ (%)	*p*-value
People in other countries				
None	1 (1)	3 (3)	2	0.231
A little	2 (2)	1 (1)	−1	
Moderately	8 (8)	10 (11)	3	
A lot	81 (83)	70 (75)	−8	
I don’t know	6 (6)	9 (10)	4	
Response	Pre-course survey (n = 96)	Post-course survey (n = 89)	Percent Δ (%)	*p*-value
Future generations				
None	0 (0)	1 (1)	1	0.132
A little	1 (1)	1 (1)	0	
Moderately	7 (7)	0 (0)	−7	
A lot	75 (78)	74 (83)	5	
I don’t know	13 (14)	13 (15)	1	
Do you think climate change can affect human health?—No. (%)				
Response	Pre-course survey (n = 104)	Post-course survey (n = 95)	Percent Δ (%)	*p*-value
Yes	104 (100)	95 (100)	0	1.000
No	0 (0)	0 (0)	0	
I don’t know, I’m not sure	0 (0)	0 (0)	0	
How important do you think it is for individuals to understand how climate change affects health?—No. (%)				
Response	Pre-course survey (n = 104)	Post-course survey (n = 95)	Percent Δ (%)	*p*-value
Very important—It is very important for everyone to understand the health risks posed by climate change so that they can take the necessary precautions and protect themselves.	94 (90)	89 (94)	4	0.364
Important—Being aware of how climate change impacts health helps people make informed decisions about their well-being and that of their communities.	9 (9)	5 (5)	−4	
Neutral—While it is beneficial for people to know about the health impacts of climate change, there may be other more pressing concerns.	0 (0)	1 (1)	1	
Not very important—While awareness of the health impacts of climate change is helpful, it may not be a priority for everyone.	1 (1)	0 (0)	−1	
Not important—People do not need to understand how climate change affects health because the impacts are minimal.	0 (0)	0 (0)	0	

## Data Availability

The raw data supporting the conclusions of this article will be made available by the authors on request.

## Abbreviations

The following abbreviations are used in this manuscript:

DR	Dominican Republic
CWP	Columbia World Project
UNIBE	Universidad Iberoamericana
CUIMC	Columbia University Irving Medical Center
GCCHE	Global Consortium on Climate and Health Education

## Appendix A

**Table A1:** Course Curriculum.

**Module 1–2: Climate Change, Human Health, and Community Resilience**
Learning Objectives: Explain the general mechanism of the greenhouse effect and describe how human activities, mainly the combustion of fossil fuels, are exacerbating this natural phenomenon Identify relevant climatic changes in the Dominican Republic (e.g., flooding, extreme heat, sea level rise) Describe major health outcomes associated with climate events, including both direct and indirect impacts, and their mechanisms Explain why some individuals and communities are more vulnerable to the health impacts of climate change Define “resilience” as it relates to climate change and the role of individuals in increasing the resilience of a community
**Module 3–4: Climate Change and Extreme Heat**
Learning Objectives: Explain how exposure to extreme heat has changed in the Dominican Republic and projections for the future Identify who is at risk of heat-related illness and why (personal, home, work, and community risk factors) Categorize signs and symptoms of heat-related illness by their respective severity and learn the appropriate actions to take if these symptoms are recognized Identify behavioral strategies and resources in the local community to protect community members during extreme heat events
**Module 5: Climate Change and Degraded Air Quality**
Learning Objectives: Describe the current air quality in Santo Domingo and identify common sources of air pollution Explain how the burning of fossil fuels contributes to climate change and air pollution Identify health impacts associated with exposure to degraded air quality Identify ways to monitor air pollution in your community Describe how to protect health in the setting of degraded air quality (protective measures at an individual, household, and community level)
**Module 6: Vector-borne Disease**
Learning Objectives: Define vector-borne disease and describe the influence of climate change on vector-borne diseases Identify important vector-borne diseases impacting the Dominican Republic and future projections (Dengue, Zika, and Chikungunya) Identify risk factors (personal, community, occupational, and environmental) for vector-borne disease Describe the core signs and symptoms of vector-borne diseases (Dengue, Zika, and Chikungunya) Identify behavioral strategies and resources in the local community to protect community members from vector-borne disease
**Module 7: Extreme Weather Events**
Learning Objectives: Explain the influence of climate change on the frequency and types of extreme weather events impacting the Dominican Republic and future projections Identify populations vulnerable to the effects of extreme weather events (children, elderly, disabled, low-resource) Describe the intersectionality between extreme weather events and other climate-related health impacts (vector-borne disease, etc.) Recommend community actions applicable to hurricane and flood preparedness and response (evacuation plans, identification and outreach to special populations, etc.)
**Module 8: Community Mental Health**
Learning Objectives Explain the impact of climate change on individual and community mental health Describe the current burden of mental health disorders in the DR Describe the psychological first aid framework Utilize psychological first aid principles to support community mental health

**Table A2:** Original Spanish Pre- and Post-Course Survey Questions.

**Climate Change Awareness**	
*Spanish*	*English*
¿Crees que el cambio climático es real?	Do you believe that climate change is real?
¿Qué tan preocupado estás por el cambio climático?	How concerned are you about climate change?
¿Qué significa “cambio climático” para ti? (Marca todos los que apliquen)	What does “climate change” mean to you? (Check all that apply)
¿Crees que el cambio climático está afectando a tu comunidad?	Do you think climate change is affecting your community?
**Climate Change Literacy**	
*Spanish*	*English*
¿Qué impactos del cambio climático son relevantes en la República Dominicana? (Marca todos los que apliquen)	What climate change impacts are relevant to the Dominican Republic? (Check all that apply)
Si el cambio climático es real, ¿cuándo crees que sucederá?	If climate change is real, when do you think it will happen?
Asumiendo que el cambio climático está ocurriendo, ¿qué crees que lo está causando?	Assuming climate change is occurring, what do you think is causing it?
¿Qué tanto crees que el cambio climático afecta a los siguientes?	How much do you think climate change affects the following?
¿Crees que el cambio climático puede afectar la salud humana?	Do you think climate change can affect human health?
Si respondiste que si a la pregunta anterior, ¿qué problemas de salud crees que podrían ser influenciados por el cambio climático? (Marca todos los que apliquen)	If you answered yes to the previous question, which health problems do you think could be influenced by climate change? (Check all that apply)
¿Qué tan importante crees que es para los individuos entender cómo el cambio climático afecta la salud?	How important do you think it is for individuals to understand how climate change affects health?
**Communication and Response Skills**	
*Spanish*	*English*
¿Qué tan confiado estás en que puedes protegerte a ti, a tu familia y a tu comunidad de problemas de salud relacionados con el clima?	How confident are you that you can protect yourself, your family and your community from climate-related health problems?
¿Qué tan confiado te sientes al hablar con otros sobre cómo el cambio climático afecta su salud?	How confident do you feel talking to others about how climate change affects their health?
¿Qué tan confiado te sientes en tu capacidad para identificar a individuos y poblaciones en riesgo de efectos en la salud relacionados con el clima?	How confident do you feel in your ability to identify individuals and populations at risk for climate-related health effects?
¿Qué tan confiado te sientes en tu capacidad para comunicar acciones que los miembros de la comunidad pueden tomar para prevenir los efectos en la salud relacionados con el clima?	How confident do you feel in your ability to communicate actions that community members can take to prevent climate-related health effects?
